# Multifactor assessments to determine the overall performance of supercritical fluid extraction from *Gynura procumbens* essential oil

**DOI:** 10.1038/s41598-022-16773-4

**Published:** 2022-08-22

**Authors:** Sitinoor Adeib Idris, Masturah Markom, Norliza Abd. Rahman, Jarinah Mohd Ali

**Affiliations:** 1 School of Chemical Engineering, College of Engineering, Universiti Teknologi MARA, 40450 Shah Alam, Selangor Malaysia; 2 Chemical Engineering Program, Faculty of Engineering & Built Environment, Universiti Kebangsaan Malaysia, 43600 Bangi, Selangor Malaysia; 3 Research Centre for Sustainable Process Technology (CESPRO), Faculty of Engineering & Built Environment, Universiti Kebangsaan Malaysia, 43600 Bangi, Selangor Malaysia

**Keywords:** Chemical engineering, Chemical engineering, Chemical safety

## Abstract

*Gynura procumbens* is a medicinal herb that contains bioactive compounds that can relieve coughs and prevent liver cancer. Supercritical fluid extraction (SFE) was suggested as one of the techniques that can be used to extract the valuable compounds from the *G. procumbens*. SFE was widely applied in extracting medicinal ingredients from herbs. However, most of them were performed only at the laboratory scale. Moreover, study to increase the yield performance, economic studies and safety assessments of the SFE process were also performed; however, these tests were conducted individually. Moreover, to date, there is no integration study between all the factors stated for determining the overall performance of SFE with herbs specifically *G. procumbens*. The integration between all the factors is beneficial because the data on the overall performance can assist in developing the SFE process with *G. procumbens* at the pilot or industrial scale. Therefore, this study incorporated a multifactor approach to measure the overall performance of the SFE process towards *G. procumbens* by using a rating and index approach. A summary of factors, such as the solubility of *G. procumbens* in CO_2_, operational cost and safety assessment elements, were taken into consideration as the main influences that determine the overall performance index of this study. *I*_*performance*_ or overall performance of SFE from *G. procumbens* was successfully assessed and compared with response surface methodology (RSM). Overall, the results from *I*_*performance*_ exhibit satisfactory solubility values when compared to the optimized value from RSM when considering the lowest operational costs in the safest SFE environment.

## Introduction

Supercritical CO_2_ extraction is usually applied to extract valuable compounds, including bioactive compounds from plant structures such as leaves, seeds, fruits and roots^1–3^. In Malaysia, the process of extracting herbs is rapidly developing. This was highly initiated in 2011 during the NKEA agriculture, and in one of the EPP projects, 18 types of herbs were chosen for further development. According to Dionysia ^4^, *Gynura procumbens* was the substance least used by traditional medicinal practitioners. *G. procumbens,* which is easily found in the tropical forests of Malaysia, Thailand and Indonesia, is an herb that contains useful compounds that can be used to relieve coughs, reduce blood glucose levels and prevent and treat liver cancer^5^. The herb is consumed raw as a salad or ‘ulam’ and can be applied topically. Moreover, *G. procumbens* contains flavonoids, saponins, tannins, and steroids, which all have potential as antioxidants^6^. The extracts contain medicinal ingredients, such as kaempferol 3-O-rutinoside, which can treat hypertension^7^; kaempferol, which is an anti-inflammatory^8^; and quercetin 3-o glucoside, which can treat diabetes^9^. Clinical studies were rigorously performed on the extracts, and all of the extracts were obtained by applying conventional and traditional techniques of extraction, such as solvent extraction using ethanol, methanol and water and hot and cold maceration techniques^10^. SC-CO_2_ extraction with ethanol–water has yet to be used in extracting valuable compounds from this herb.

The supercritical solvent used in SFE, which is CO_2_, is efficient in extracting non-polar components, such as terpenes and alkaloids, from plant samples^11^. Since the targeted compounds are antioxidants that are semi-polar, a co-solvent is introduced to improve the selectivity. For example, a semi-polar co-solvent, such as ethanol, was introduced to enhance the overall quality of the yield^12^. To date, many researchers have incorporated water into the co-solvent to further enhance the extraction process^13–16^. Water can modify the structure of the matrix inside the sample due to its higher viscosity compared to that of CO_2_ and ethanol and its lower solubility in CO_2_ compared to that of ethanol^17^.

Simulation of the extraction curve has been performed rigorously to predict the effect of parameters on the extract yields and to determine the optimum parameter for the best yield using various mathematical model approaches^18^. Sovová and Stateva^19^ reviewed types of mathematical models for SFE kinetics. According to them, there are five types of models, including the following: mass balance for solute, extraction steps and their characteristic times, a one-stage model, a model based on the complex structure of plant particles and a model for the SFE of mixtures^19^. Table 1 summarizes the reported study on applying a mathematical model in fitting the experimental data for the SFE-co-solvent process. The model was then analysed to determine the best operating conditions that produce the highest yield. According to Table 1, the pure ethanol and ethanol–water mixture was the most commonly used co-solvent for SFE. The broken and intact cell model (BIC) model was the most fitted model in fitting the experimental data. The BIC model is usually applied to a mechanically damaged cell sample due to the sample preparation^20^. The most important parameter for the BIC model was the initial fraction of easily accessible solute, G, in which a value between 0 and 1 was obtained^21^.

**Table 1:** Previous study on the mathematical models applied to SFE with a co-solvent for plants.

Plant	Co-solvent	Mathematical model	Results	References
*Sumac seeds*	Ethanol	Differential mass balances and shrinking core model (SC)	Both mathematical models can well predict the behaviour of the process and fit the data	^22^
Leaves and stems of Syn*adenium grantii*	Ethanol	First order empirical model	The model fit with the experimental curve	^11^
*Castanea sativa*	Ethanol	Empirical model^23^, logistic model^24^, desoprtion model^25^, and broken and intact cell model (BIC)^26^	The BIC model was the most fitted with the extraction curve followed by the logistic and desoprtion model. The empirical model was the least fitted	^27^
*Cannabis* hybrid flower	Ethanol	Broken and intact cell model (BIC) model^26^	The model fit with the experimental curve	^28^
*Eremanthus erythropappus*	Ethanol	Broken and intact cell model (BIC) model^26^	The model fit with the experimental curve	^29^
*Spirulina platensis*	Ethanol Water Ethanol–water	Differential mass balance model	The model fit with the experimental curve	^30^
*Hypericum caprifoliatum*	Ethanol Water Ethanol–water	Simplified broken and intact cell model (BIC) model^31^, differential mass balance model^32^ and desorption model^25^	The differential mass balance model was the most fitted with the extraction curve	^33^
*Phyllanthus niruri*	Ethanol–water	Modified Sovová model^34^	The model fit with the experimental curve	^17^

The experiment that obtained the highest yield under optimum conditions was also considered to achieve the best performance by the SFE process. The values of the optimum conditions can be evaluated by using a statistical tool, such as response surface methodology. This tool enables the user to choose the operating conditions, which can be optimized to obtain the highest yield. Moreover, the tool offers a good package that can provide a good design and analysis of the process by applying the statistical significance of all the factors used with analysis of variance. In addition, an artificial neural network is another piece of computing system that has been fully utilized in simulating the results of SFE. The network simulates the results by following the way the human brain analyses and processes information. This software is highly used due to its advantages, including that no thermophysical understanding of the SFE process is needed to conduct the simulation. In addition, previous knowledge about the neural network is not needed^35^. The ANN structure consists of a multi-layer, fully connected input layer, hidden layer, and output layer. The sensory data (experimental data) fed to the network is interpreted by the machine perceptron, which labels the input data and identifies the numerical patterns.

Table 2 shows the studies that have applied techniques to achieve the highest yield for the SFE process. Several studies have reported the application of two types of extraction techniques to achieve high yields in contrast to the process with SFE alone. For example, when extracting *Caryocar Brasiliense,* clove buds *and Dipteryx alata*, cold pressing was used together with SFE to achieve higher yield than that of SFE alone^36–38^. Economic evaluation has been conducted previously, in which the cost of manufacturing (COM) was determined. The calculated COM was compared between the SFE plant at the laboratory scale, pilot scale and industrial scale for production^37–39^. There is also a study focusing on economic assessment to evaluate the feasibility of the SFE process for the purpose of scale-up^17^. Moreover, there were also assessments of the safety in conducting the SFE process^40^. Most of these assessments were done separately and independently. None of the evaluations were systematically integrated to measure the overall performance of the SFE process.

**Table 2:** Techniques to achieve high performance in the SFE process.

Sample	Performance enhancement technique	Results	Observation	References
*Caryocar brasiliense*	SFE + cold pressing	Can achieve an 8 times higher yield than that of SFE	Suitable for sample with higher lipid content	^36^
Clove buds	SFE + cold pressing + economic evaluation	Can obtain a 5 times higher yield extract than that of SFE	The cost of manufacturing (COM) for SFE + cold pressing is lower than that for the SFE system	^37^
*Dipteryx alata*	SFE + cold pressing + economic evaluation	Yield a higher yield by 31% over SFE	The cost of manufacturing (COM) for SFE + cold pressing is lower than that of the SFE system	^38^
*Scrophularia striata Boiss*	SFE + ultrasonic + economic evaluation	The performance of SFE was better with the ultrasonic treatment	The energy cost is lower when SFE is combined with ultrasonic treatments	^41^
*Eugenia pyriformis*	SFE + co-solvent + economic evaluation	An addition of up to 5% (w/w) of ethanol to SFE resulted in a higher yield	The production costs decrease when the SFE was added with a co-solvent	^42^
*Capsicum frutescens*	SFE + economic evaluation	SFE at a larger scale is better	COM was performed for three different scale of SFE	^39^
Rachig ring and glass beads	SFE + mathematical model + safety assessment	The valve opening needs to be control to produce an optimum depressurization	The mathematical model is used for simulations for the depressurization processes, which were involved with the temperature and pressure of SFE	^43^
*Benzoic acid*	SFE + economic analysis + safety assessment + mathematical model + artificial intelligence	–	The increase in pressure and temperature caused the economic and safety performance to decrease for SFE	^44^

To have an idea of the overall performance of SFE, a statistical report to measure the performance is needed. This study tends to consider the incorporation of multiple factors, such as yield, economic factors, and safety, in evaluating the overall performance of the SFE process of *G. procumbens* during the operation of the system. The results will be represented as an index that will act as an indicator to determine the overall performance of the SFE process.

## Materials and method

### Sample and chemicals

*Gynura procumbens* was obtained from a local company, HERBagus Trading Sdn. Bhd which is located in Kepala Batas, Pulau Pinang, Malaysia. The sample received was cleaned and dried in open air for two days followed by drying using an oven at 50 °C until the total moisture content in the sample was less than 10%. The sample was then sieved to obtain a size of 2.0 mm and was stored at room temperature until use. The chemicals used were carbon dioxide (99.5% purity), which was purchased from Alpha Gas (Malaysia), and ethanol (99.8% purity), which was purchased from QRec (Malaysia).

### Supercritical fluid extraction

A laboratory scale SFE unit that was designed and installed by a previous researcher was used for this study^34^. Chiller was first switched on to let the CO_2_ cool to − 4 °C to let the gas change to the liquid phase before being pumped to the SFE system. Three grams of *G. procumbens* ground leaves was inserted into the pressure vessel (H/D = 8). The oven was switched on, and the temperature was set to the designated operating temperature. The pressure on the back-pressure regulator was also set to its designated operating pressure. The combination factors for the SFE run for this study are shown in Table 3. The design of this experiment was performed by using a response surface method by Design Expert^®^ (Stat-Ease, USA). Central composite design was utilized with three factors, which were the temperature (°C), pressure (MPa) and water content in ethanol (%). The response was fixed to the overall yield (g/g %) and solubility of *G. procumbens* in CO_2_ (g/g %). The α value was chosen as k > 5 with a value of 1.32. There are 20 runs, and the CCD positions of 8 factorial points, 6 axial points and 6 centre points are shown in Table 3.

**Table 3:** CCD experimental design for the SFE of *G. procumbens.*

Run	Factor 1	Factor 2	Factor 3	CCD position
	A: Pressure (MPa)	B: Temperature (°C)	C: Water content in ethanol (%)	
1	24	60	30	Factorial
2	24	70	30	Factorial
3	21	58	20	Axial
4	18	70	30	Factorial
5	24	60	10	Factorial
6	21	72	20	Axial
7	25	65	20	Axial
8	21	65	33	Axial
9	21	65	20	Center point
10	18	60	30	Factorial
11	21	65	20	Center point
12	24	70	10	Factorial
13	18	70	10	Factorial
14	18	60	10	Factorial
15	21	65	7	Axial
16	17	65	20	Axial
17	21	65	20	Center point
18	21	65	20	Center point
19	21	65	20	Center point
20	21	65	20	Center point

### Optimization using the overall performance index

#### Economic evaluation

To evaluate the economic element in the SFE of *G. procumbens*, the methodology by Turton et al. ^45^ was referred to when estimating the operational costs. Initially, they presented 3 categories to estimate the cost of manufacturing (*COM*), including the following: direct costs, fixed costs, and general expenses. Since this study focuses on calculating the operational cost, which is tabulated in Table 4, the fixed costs were not taken into consideration. The cost of waste treatment was also excluded since solid waste from SFE can be added to soil for the decomposition process. Therefore, the operational cost consists of the cost of raw materials (*CRM*), cost of utilities (*CUT*) and labour cost (*COL*). The economic parameter that was used to estimate the operational costs (*OC*) is also shown in Table 4. Therefore, the estimation of *OC* can be simplified from Turton et al. ^45^ to Eq. (1) as follows:

1 $$OC = 2.73 \times COL+1.23\times (CUT+CRM)$$

where *OC* is in units of RM/year.

**Table 4:** The description of each category in direct costs.

Cost	Description	Unit	Price	References
Raw materials costs (*CRM*)	Price of the *G. procumbens* sample	RM/kg	70	HERBagus Trading, Malaysia
	Transportation and sample preparation costs	–	–	–
	Price of ethanol	RM/bottle(2.5 l)	86	BT Science Sdn Bhd
	Price of CO_2_	RM/cylinder (30 kg)	224	Alpha Gas Solution Sdn. Bhd
Utilities cost (*CUT*)	Electricity CO_2_ pump Co-solvent pump Back-pressure regulator Oven Chiller Lamp, fan and air-conditioning unit	sen/kWh	0.365	Tenaga Nasional Berhad
Labour cost (*COL*)	One operator Graduate research assistant (GRA)	RM/month	1800	Ministry of Higher Education, Malaysia

Table 4 shows the description of each category in direct costs. In the raw materials costs (*CRM*), the price of CO_2_ dominates the costs. For utility costs (*CUT*), the use of electricity mostly originates from the equipment in the SFE system, as listed in Table 4.

#### Safety assessment

1. First stage of the safety assessment.
  Two objectives in evaluating the first stage were used to identify the hazard when conducting the SFE *G. procumbens* experiments and to classify the risk of hazards that can occur (light, moderate, intermediate, heavy, and severe). An analysis of the most hazardous equipment for SFE was conducted. After that, the potential of the second effect from the main scenario analysis was performed.
2. Second stage of safety assessment

A methodology by a previous researcher was used when evaluating the quantitative analysis of safety from SFE of *G. procumbens*^46,47^. This study used a mixture of ethanol–water as the co-solvent for the SFE process at a ratio of 10–30% v/v water–ethanol. Therefore, the risk of using the mixed co-solvent was estimated by identifying the boiling point (*t*_*b*_), flash point (*f*_*p*_) and Hansen solubility value (δ) for each of the ratios of water in ethanol. Equation (2) was applied to estimate *t*_*b*_. Equation (3) was used to determine the *f*_*p*_*.*

Equation (2) was used to determine the value of *t*_*b*_ for the mixture. *x*_*1*_ is the mol fraction of solvent 1, *x*_*2*_ is the mol fraction of solvent 2, *t*_*b1*_ is the boiling point for solvent 1 and dan *t*_*b2*_ is the boiling point for solvent 2.

2 $${t}_{b}={x}_{1}{t}_{b1}+{x}_{2}{t}_{b2}$$

To estimate the flash point of the mixture, Eq. (3) was used.

3 $$\sum_{i=1}^{2}\frac{{x}_{i}{\gamma }_{i}{P}_{i,sat}}{{P}_{i,sat FP}}=1$$

where *x*_*i*_ is the mol fraction, ${\gamma }_{i}$ is the activity coefficient, ${P}_{i,sat}$ is the vapour pressure at T and ${P}_{i,sat FP}$ is the vapour pressure at the flash point.

Equation (4) was used to determine the Hansen solubility value, whereby *x*_*1*_ is the mol fraction of solvent 1, *x*_*2*_ is the mol fraction of solvent 2, *D*_*1*_ is the solubility for solvent 1 and *D*_*2*_ is the solubility for solvent 2.

4 $$\delta ={x}_{1}{D}_{1}+{x}_{2}{D}_{2}$$

To estimate the Chemical Safety Total Score (*CSTS*), several factors need to be taken into consideration, and these factors are listed in Table 5. The factors that need to be determined are flammability, toxicity, reactivity, and explosiveness parameter. The equations used are displayed in Table 6. Then, the parameter was summed up as in Eq. (9) to obtain the value of *CSTS*.

**Table 5:** Logistic function for determining the parameter for each factor.

Factor	Parameter	Logistic function	Equations
Flammability, *S*_*FL*_	Flash point	${S}_{FL}=100\times \left(1-\left(\frac{1}{1+3.37{e}^{-0.024x}}\right)\right)$	(5)
Toxicity, *S*_*TX*_	Threshold limit values (TLV) for short-term exposure limit (STEL)	${S}_{TX}=100\times \left(1-\left(\frac{1}{1+403.4288{e}^{-0.012x}}\right)\right)$	(6)
Reactivity, *S*_*R*_	*National Fire Protection Association* (NFPA)	${S}_{R}=100\times \left(\frac{1}{1+270.43{e}^{-2.8x}}\right)$	(7)
Explosiveness, *S*_*EXP*_	Lower and Upper Explosiveness Limit (%UEL–%LEL)	${S}_{EXP}=100\times \left(\frac{1}{1+1096.63{e}^{-0.14x}}\right)$	(8)

**Table 6:** ANOVA by CCD design for SFE of *G. procumbens.*

Response	Yield	Solubility
Prob > F	< 0.0001	0.0028
Lack of fit	0.4441	0.0050
R-squared	0.9529	0.8621
Pred R-squared	0.8932	0.0031
Adj R-squared	0.9312	0.7380
Significant factor	A	A
	B	C
	C	AC
	AC	
Coefficient	A = 3.20 B = 1.13 C = 3.10 AB = − 0.05 AC = 2.11 BC = 0.77 A2 = – B2 = – C2 = –	A = 0.49 B = 0.03 C = 0.33 AB = 0.09 AC = 0.42 BC = − 0.16 A2 = 0.25 B2 = − 0.07 C2 = 0.28

9 $$CSTS = {S}_{FL}+{S}_{TX}+{S}_{R}+{S}_{EXP}$$

#### Overall performance index of SFE *G. procumbens*

Equation (10) was used to determine the performance index of SFE as follows:

10 $${I}_{performance}={I}_{solubility}+{I}_{cost}+{I}_{safety}$$

All of the parameters were a function of temperature (*T*), pressure (*P*) and water content in ethanol ($\omega$) and can be written as $f\left(T,P,\omega \right)$. The solubility data were taken from RSM. The data for cost can be calculated based on Eq. (1), and safety was obtained from Eq. (9).

## Results and discussion

### Regression using RSM

Both responses were successfully simulated using RSM, with yield regression using a 2-factor interaction (2FI) and solubility regression by a quadratic model. Table 6 shows the ANOVA by the CCD design and the details of the significant factor and coefficient for each factor for the regression equation.

From Table 6, Prob > F values are significant for both responses, and the values obtained were less than 0.05. Both responses were affected by the individual factors A and C and the interaction factor of A and C. The R^2^ values above 0.86 for both responses are also reasonable. Therefore, the reported research on applying CCD for the model regression for SFE was also indicated to be reasonable for use in this study^48^.

### Effect of temperature and pressure on the yield and solubility

**Figure 1:**
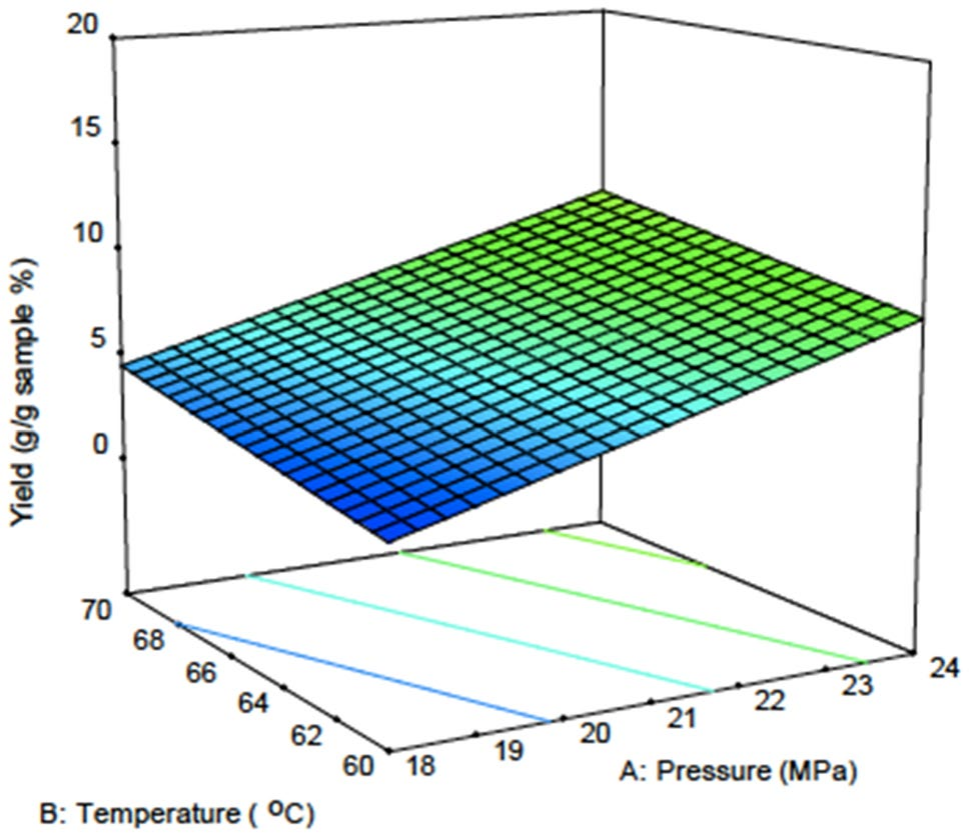
Response surface plot for yield versus temperature and pressure.

**Figure 2:**
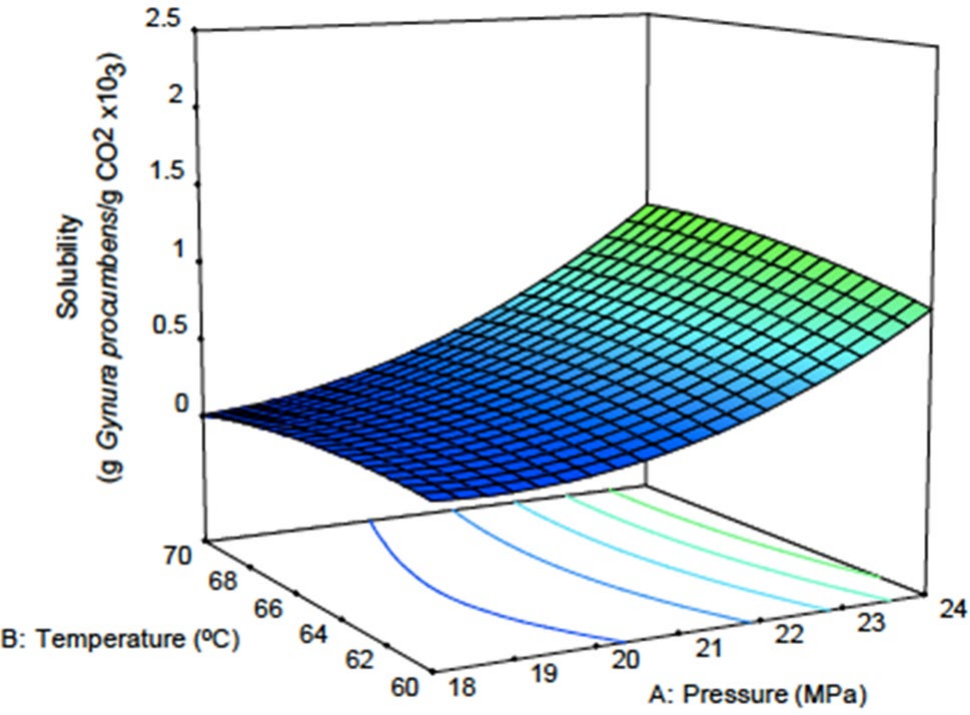
Response surface plot for solubility versus temperature and pressure.

Table 7 shows the yield and solubility results from the experiment. Figures 1 and 2 show the response surface plot for the effect of temperature and pressure on the yield and solubility. Figure 1 shows that at 60 °C, the yield increased with pressure and improved at a higher temperature of 70 °C. This shows that the contact between the solute and solvent is better with pressure. The density of CO_2_ depends on the pressure and greatly affects the solubility of the solute in the CO_2_^49^. Figure 2 clearly shows that the contour density is greatly affected by pressure compared to temperature for the solubility of *G. procumbens* in CO_2_. In other research, it was reported that when higher pressures are used for SFE, the effect of temperature on density is less noticeable, and the dominant factor is the vapour pressure^50^. When lower pressures are used, the change in temperature is more pronounced, and the process is dominated by the solvent density^51^_._ From here, we can see that there are variations in the solvation power of supercritical CO_2_ under different operating conditions.

**Table 7:** The results obtained from the CCD design of the experiments.

Run	Factor 1	Factor 2	Factor 3	Yield	Solubility
	A: Pressure (MPa)	B: Temperature (°C)	C: Water content in ethane (%)	(g/g %)	(g *G. procumbens/*g CO_2_) × 10^3^
1	24	60	30	12.9	2.4
2	24	70	30	15.9	2.35
3	21	58	20	4.05	0.2
4	18	70	30	6.26	0.1
5	24	60	10	3.73	0.3
6	21	72	20	7.87	0.2
7	25	65	20	12.74	1.0
8	21	65	33	11.8	0.8
9	21	65	20	4.9	0.4
10	18	60	30	2.23	0.5
11	21	65	20	5.61	0.3
12	24	70	10	4.5	0.9
13	18	70	10	2.47	0.3
14	18	60	10	2.35	0.1
15	21	65	7	3.18	0.8
16	17	65	20	2.9	0.5
17	21	65	20	5.8	0.4
18	21	65	20	6.88	0.5
19	21	65	20	6.9	0.6
20	21	65	20	7.5	0.3

### Effect of water content in ethanol on yield and solubility

**Figure 3:**
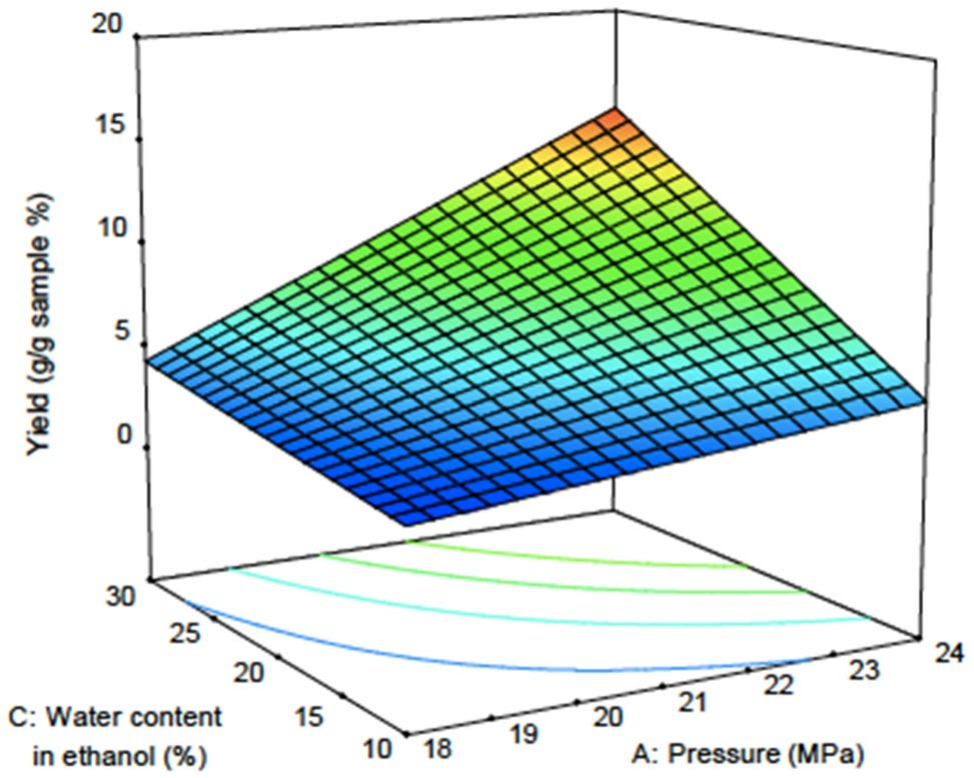
Response surface plot for yield versus pressure and water content in ethanol.

**Figure 4:**
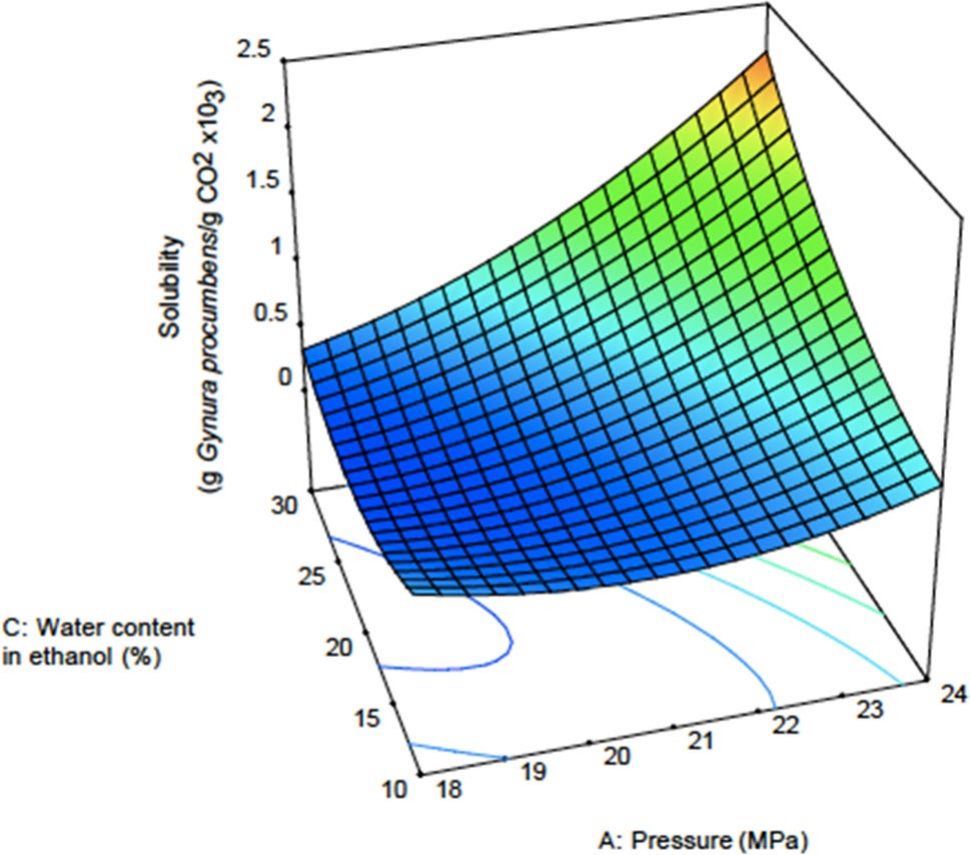
Response surface plot for solubility versus pressure and water content in ethanol.

Figures 3 and 4 show the response surface plot for the effect of water content in ethanol on the yield and solubility at different pressures and water contents in ethanol. The effects of water content in ethanol can clearly be seen at the highest pressure of this experiment, which is 24 MPa. This is because of the enhancement of the solute solubility to the solvent, and this enhancement was influenced by the amount of water inside the ethanol^14^. When in contact with the sample, water can alter the sample matrix. A previous study reported that, compared to ethanol, water can better penetrate through the cell wall^52^. Water can also extract more lignin compounds in the secondary cell wall than in the layer between the cells^52^. This is due to the higher density of water compared to ethanol (Table 8). When this happens, the hole at the surface of the wall opens widely, causing the amount of lignin inside the sample to decrease. Moreover, a report from^52^ mentioned that carbon dioxide created an acidic environment when reacting with water. This triggers hemicellulose and lignin degradation on the primer cell wall. Therefore, the cell wall is no longer intact because the primer cell wall has been destroyed. Resistance towards the surface tension is also zero. Therefore, more CO_2_ can penetrate inside the cell to extract solute located at the secondary cell wall.

**Table 8:** Density of water, ethanol, and the ethanol–water mixture at different temperatures and pressures.

Temperature (°C)	Pressure (MPa)	Density (g/ml × 10^–3^)				
		Water	Ethanol	Ethanol–water (70% v/v)	Ethanol–water (80% v/v)	Ethanol–water (90% v/v)
65	25	991.17	773.97	815.53	795.68	789.11
	21	989.51	770.51	812.59	780.99	783.22
	17	987.83	766.77	809.48	789.13	779.69

Figure 3 shows that the highest extract was obtained at 24 MPa and was greater when the water content was increased from 10 to 30% inside ethanol. At low pressure, the water content does not have any effect on the yield obtained. Figure 4 clearly shows that the solubility value does not increase with increasing water content in ethanol. However, effective extraction occurred at the highest pressure of 24 MPa.

### Operational cost (*OC*)

**Figure 5:**
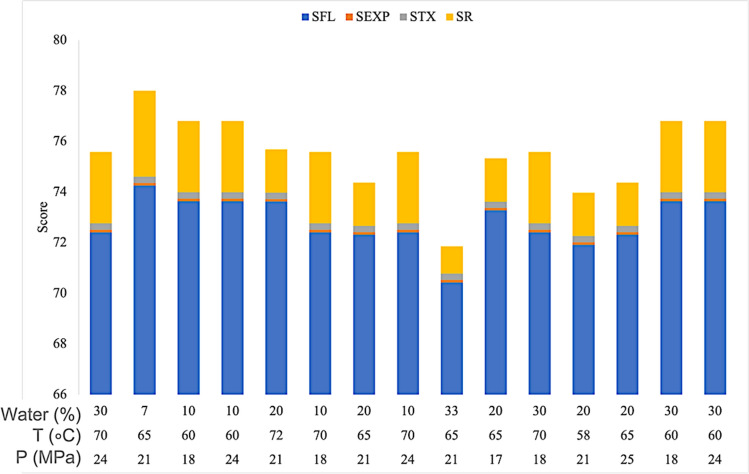
Individual factors in the *CSTS* score at each parameter for the SFE of *G. procumbens.*

**Figure 6:**
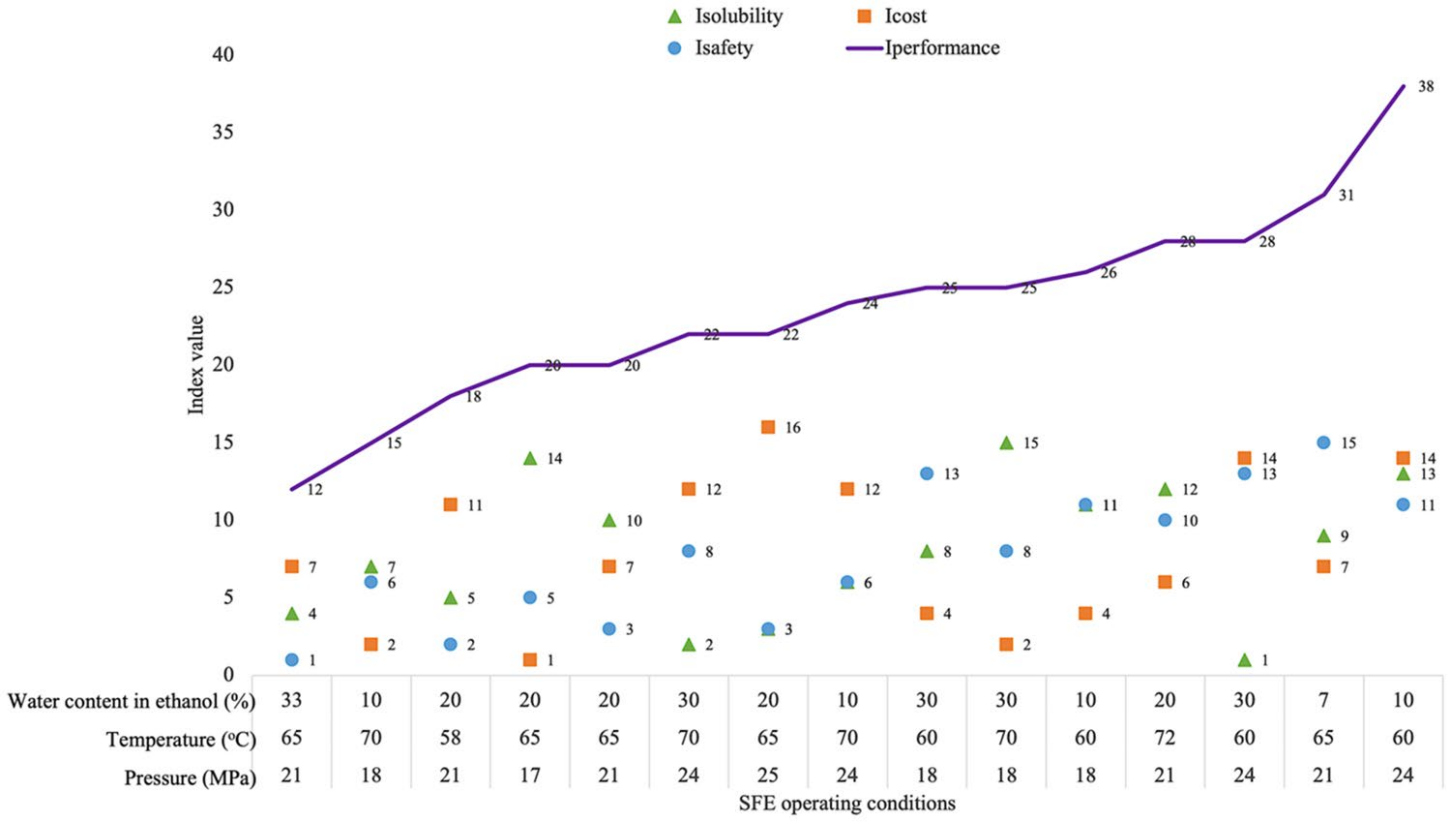
Overall performance index, *I*_*performance,*_ for SFE *G. procumbens.*

Figure 5 shows the fractions of the operational costs for the SFE of *G. procumbens*. The highest cost was the utility cost, which was 58% of the total, followed by labour cost (22%) and raw materials cost (20%). The highest contributor to the utility cost came from the electricity cost of the chiller, which utilized 13.82 kW, whereas other equipment, such as pumps, ovens and back-pressure regulators, contributed less than 1 kW each. Attard et al. (2015) reported that the same findings that *CUT* is the majority of the operational cost of conducting SFE^53^. The *COM* was further analysed to determine the distribution of each element (Fig. 6). The price of CO_2_ dominates the cost of *CRM*, with nearly 80%, followed by ethanol (20%). Previous research reported that the highest distributor in *CRM* was the cost of the sample. This is due to the sample supplier charging a high price. Moreover, a previous study was concerned with the higher production rate; therefore, a sample with a high mass was needed for extraction, resulting in higher cost^54^. The total operational costs were calculated for each parameter of the SFE *G. procumbens*. Then, it was further ranked to give the *I*_*cost*_ value.

### Safety assessment

The main equipment in the SFE process that involve risks and hazards are listed in Table 9. Three pieces of equipment with the potential of experiencing overpressure were the CO_2_ pump, co-solvent pump, and pressure vessel. However, the following pieces of equipment involve a risk of boiling liquid expanding vapour exploration (BLEVE): CO_2_ storage tanks and pressure vessels. The types of hazards for each of the main equipment are also listed in the table. In this study, chemical hazards can occur with all the main equipment. This is because each of the equipment involves the solvent, which is the chemical in this process.

**Table 9:** Risk for each of the main equipment in SFE of *G. procumbens.*

Main equipment	Risk	Type of hazard
CO_2_ storage tank	BLEVE	Chemical hazard Thermodynamic hazard Biological hazard
CO_2_ pump	Overpressure	Chemical hazard Biological hazard
Co-solvent pump	Overpressure	Chemical hazard
Pressure vessel	BLEVE Overpressure	Chemical hazard Thermodynamic hazard Mechanical hazard

Table 10 shows the potential of secondary scenarios after the main scenario that can happen due to BLEVE. BLEVE is an explosion due to the failure of the pressure vessel, which is filled with liquid and is unable to withstand a temperature greater than the boiling point temperature at atmospheric pressure. According to Table 10, when overpressure occurs, it acts as a vector for the main scenario, after which a secondary potential scenario can form, such as a flash fire and toxic release. According to a previous study, studies on BLEVE were mostly performed on LPG and propane^55^. The effect of CO_2_ is still under review and can be further explored^56^.

**Table 10:** Secondary potential scenario from the BLEVE.

Main scenario	Vector	Secondary potential scenario
BLEVE	Overpressure	Flash fire Pool fire Jet fire Fire ball Vapour cloud explosion, (VCE) BLEVE Toxic release

The second safety assessment was conducted to determine the chemical safety total score (*CSTS*) by calculating each of the factors. According to Fig. 5, the *CSTS* scores were found to be the highest at 7% water content in ethanol. This is because the volume of ethanol was the largest. The highest portion for each *CSTS* score originated from the *S*_*FL*_ score. This shows that the water content in ethanol does influence the flammability factor of the SFE process. *S*_*EXP*_ and *S*_*TX*_ do not exhibit any variance at different parameters. However, *S*_*R*_ has a small influence on *CSTS*. The *CSTS* value was then ranked to determine the safety index for this study.

### Overall performance index

The overall performance index for this study was determined by using Eq. (10). Individual indices including solubility, cost and safety were summed to obtain the total index, *I*_*performance*_. Figure 6 shows the overall performance of SFE *G. procumbens* at each parameter of the study. In addition, the individual index value was also shown to illustrate the factor that distributed the most for each performance. To choose the best performance from the parameters listed, the aim was to obtain the highest solubility at the lowest operational cost and in the safest environment. From Fig. 6, it can be concluded that the best *I*_*performance*_ was obtained at 21 MPa, 65 °C and 33% water content in ethanol (v/v).

Table 11 shows the optimum results obtained from the RSM method and the best performance from the *I*_*performance*_ method. Different parameter values were obtained from both. This was because the assessment for RSM does not consider the operating cost and safety factor when determining the optimum conditions, in which *I*_*performance*_ integrates multiple factors to determine the best value. Overall, the results from *I*_*performance*_ exhibit satisfactory solubility values when compared to the optimized value from RSM when considering the lowest operational costs in the safest SFE environment.

**Table 11:** Comparison of the solubility data obtained from different methods of performance.

Method of assessment	Pressure (MPa)	Temperature (°C)	Water content in ethanol (%)	Solubility (g *G. procumbens/*g CO_2_) × 10^3^
RSM	24	68.8	29.8	1.89
*I*_*performance*_	21	65	33	1.30

## Conclusion

The overall performance index method is satisfactory for evaluating the SFE operation with *G. procumbens*. The results show that the value of optimum solubility from RSM did not differ much from the value obtained by *I*_*performance*_. However, a different parameter was chosen, whereby the pressure and temperature were chosen at the centre point and the water content in ethanol was selected at 33% (v/v) for the method by *I*_*performance*_. The water content affected the process as well as the safety of the SFE process, especially the flammability factor, *S*_*FL*_. Water, when added to ethanol, altered the matrix sample and assisted the mass transfer process of solute to the solvent (CO_2_ and ethanol). The economic evaluation reported that the highest cost in operational costs (*OC*) originated from utility costs (*CUT*), and the highest contributor was from the chiller. Breakdown of the raw materials costs (*CRM*) indicates that the cost of CO_2_ dominates the expense. The results from the safety assessments towards the SFE process imply that there were 2 types of risk that can occur to the pressure vessel, including BLEVE and overpressure. Moreover, a secondary potential scenario can occur when BLEVE is further boosted by overpressure. The solubility results from *I*_*performance*_ are satisfactory compared to those from RSM. This suggests that the index method by rating of the individual factors of solubility, economy and safety was adequate to recommend the best operating conditions for the highest solubility, as well as for obtaining minimum operational costs and the safest conditions possible.

## Data Availability

The datasets used and/or analysed during the current study available from the corresponding author on reasonable request.
